# Comparative Analysis of Differentially Expressed Circular RNAs in Polarized Macrophages

**DOI:** 10.3389/fgene.2022.823517

**Published:** 2022-03-16

**Authors:** Rong-mei Zhou, Ze-hui Shi, Kun Shan, Shu-jie Zhang, Yi-han Zhang, Yu Liang, Biao Yan, Chen Zhao

**Affiliations:** ^1^ Eye Institute and Department of Ophthalmology, Eye and ENT Hospital, Fudan University, Shanghai, China; ^2^ NHC Key Laboratory of Myopia (Fudan University), Shanghai, China; ^3^ Key Laboratory of Myopia, Chinese Academy of Medical Sciences, Shanghai, China; ^4^ Shanghai Key Laboratory of Visual Impairment and Restoration, Shanghai, China

**Keywords:** macrophage polarization, THP-1 cell, next-generation RNA sequencing, circular RNA, circRNA-RNF19B circRNAs in macrophage polarization

## Abstract

Macrophage polarization is a process that macrophages exert different functions according to surrounding micro-environment. Macrophages commonly exist in two distinct subsets: classically activated M1 macrophages and alternatively activated M2 macrophages. Circular RNAs (circRNAs) are a novel class of non-coding RNAs generated by back-splicing. Thousands of circRNAs were identified in different cells and tissues. Recent studies have revealed that circRNAs play a crucial role in regulating transcriptional and post-transcriptional gene expression. However, the effects and roles of circRNAs in macrophage polarization have not been well elucidated. Here, circRNAs expression profiles were determined in human THP-1 macrophages incubated in conditions causing activation toward M1 (interferon-γ + LPS) or M2 (interleukin-4) phenotypes. Overall, 9,720 circular RNA were detected from RNA sequencing data. Compared with M2 macrophages, a total of 140 circRNAs were aberrantly expressed in M1 macrophages, including 71 up-regulated circRNAs and 69 down-regulated circRNAs. Quantitative real-time PCR (qRT-PCR) results were generally consistent with the selected differentially expressed circRNAs. Gene Ontology (GO) and KEGG pathway analyses were used to predict biological functions and potential mechanisms of the host linear transcripts of these up-regulated and down-regulated circRNAs. Furthermore, we found that the expression level of circRNA-RNF19B (circRNF19B) in M1 macrophages was significantly higher than that in THP-1 macrophages and M2 macrophages. circRNF19B expression was increased when M2 converted to M1 whereas decreased when M1 converted to M2. Knockdown of circRNF19B following the activation of THP-1 cells using interferon-γ + LPS diminished the expression of M1 macrophages markers and elevated the expression of M2 macrophages markers. In conclusion, these data suggest the involvement of altered circRNAs expression patterns in macrophages exposure to different activating conditions. Circular RNAs may play important roles in regulating macrophage polarization.

## Introduction

Macrophages are the essential components of innate immunity with remarkable plasticity and functional heterogeneity (Sica and Mantovani 2012). Macrophage polarization is a process that macrophages commonly shaped in tissues where they are able to alter their phenotype and functions in response to local micro-environmental stimulus. Two well-established polarized phenotypes are often referred to as classically activated macrophages (M1 macrophages) and alternatively activated macrophages (M2 macrophages) (Locati et al., 2020).

M1 macrophages are pro-inflammatory and antigen presenting cells, which can be typically activated by stimulation with lipopolysaccharide (LPS) alone or with interferon-γ (IFN-γ), toll-like receptor (TLR) ligands (Sica and Mantovani 2012; Locati et al., 2020) They are characterized by high antigen presentation and high expression of pro-inflammatory cytokines such as tumor necrosis factor-α (TNF-α), interleukin (IL)-1β, IL-6, IL-12, IL-23, high production of reactive oxygen species and NO, promotion of Th1 response, and robust anti-microbial and anti-tumor activity (Sica and Mantovani 2012; Orecchioni et al., 2019; Locati et al., 2020). In contrast, M2 macrophages are more heterogeneous and assumed to be induced by a variety of stimuli including the Th2 cytokines IL-4 and IL-13, the anti-inflammatory IL-10, M-CSF, glucocorticoids, immune complexes, and apoptotic cells. They are generally identified by high production of IL-10, TGF-β and low production of IL-12 and IL-23 and up-regulation of arginase-1 (Arg-1), mannose receptors (CD206), and scavenger receptors (CD163). Functionally, M2 macrophages have efficient phagocytic activity, scavenge debris and apoptotic cells, eliminate parasites, dampen inflammation, promote angiogenesis and take part in tissue repair and wound healing (Sica and Mantovani 2012; Orecchioni et al., 2019; Locati et al., 2020).

Due to the diversity of macrophages function, macrophage polarization is widely involved in the regulation of various disease such as infection, metabolic diseases, obesity, autoimmune diseases and tumors (Mills 2012; Zhuge et al., 2016). Thus, the investigation of molecular mechanisms underlying macrophage differentiation and polarization provides a novel perspective on macrophage-centered diagnostic and therapeutic strategies. However, the regulatory mechanisms that control the expression of the constellation of genes in macrophage responding to polarizing conditions have not been fully established.

Circular RNAs (circRNAs) are known as a novel class of noncoding RNAs (ncRNAs). They are produced by pre-mRNA back-splicing events and formed by the covalent bond linking the 3′ and 5′ ends (Kristensen et al., 2019). CircRNAs are widely expressed in mammals and show more stable properties than their corresponding linear mRNAs. Due to their capacities to sequester microRNAs (miRNAs), circRNAs play essential roles in the regulation post-transcriptional gene expression (Hansen et al., 2013; Panda 2018). In addition, increasing studies have reported that aberrant expression of circRNAs is closely associated with progression of pathologic conditions including cardiovascular diseases (Altesha et al., 2019; Wang and Liu 2020), neurological diseases (Mehta et al., 2020), diabetes (Filardi et al., 2020) and cancers (Lei et al., 2019). Emerging evidence revealed that other two kinds of ncRNAs, long non-coding RNAs and miRNAs, are important regulators of the differentiation and function of monocytes/macrophages (Kim et al., 2019; Xu et al., 2021). However, the comprehensive expression profile and the role of circRNAs in macrophage polarization still remain to be explored.

In this study, we employed next-generation RNA sequencing to document changes in the expression profile of circRNAs between two distinct polarizing THP-1 macrophages [M1 (interferon-γ + LPS) and M2 (IL-4)]. Our data identified that the expression of a number of circRNAs was altered under different polarizing conditions. More importantly, we characterized one circular RNA derived from RNF19B gene locus (has_circ_0000048), which may play a critical role in promoting macrophage polarization to the M1 phenotype. Thus, our study reveal that circRNAs are regulators of macrophages gene expression that contribute to macrophage differentiation on the basis of polarizing environmental conditions.

## Materials and Methods

### Cell Culture and Reagents

Human monocytic THP-1 cells were cultured in RPMI 1640 medium containing 10% heat inactivated fetal bovine serum (FBS, Gibco). Cells were incubated at 37°C with 5% CO2 in a humidified atmosphere. The THP-1 cells in suspension were differentiated into adherent macrophages by treating with 100 nmol/L phorbol-12-myristate-13-acetate (PMA) (Sigma) for 48 h.

### Macrophage-Polarizing Conditions

To induce the polarization of macrophages, after the cells were washed with PBS to remove the PMA, THP-1 derived macrophages were polarized into M1 macrophages by incubation with 20 ng/ml of IFN-γ (PeproTech) and 100 ng/ml of LPS (Sigma). M2 macrophage polarization was achieved by incubation with 20 ng/ml of interleukin 4 (IL-4, R&D Systems). The supernatants were collected after polarization for 48 h and adherent cells were harvested after polarization for 18 h for further analysis.

### RNA Extraction and Next-Generation RNA Sequencing

Total RNAs of M1 and M2 macrophages were isolated using mirVana miRNA Isolation Kit (Ambion) according to the manufacturer’s instruction. The concentration and purity of RNAs were determined by NanoDrop ND-1000 (Thermo Fisher Scientific, Waltham, MA). The ratio of OD260/OD280 was between 1.8 and 2. RNA Integrity was determined by Agilent 2100 Bioanalyzer (Agilent Technologies, Santa Clara, CA, United States). The samples with RNA Integrity Number (RIN) ≥ 7 and 28S/18S ≥ 0.7 were subjected to subsequent analysis.

The libraries were constructed using TruSeq Stranded Total RNA with Ribo-Zero Gold (RS-122-2301, Illumina) based on the manufacturer’s manuals. In short, total RNAs of each sample were treated with Ribo-Zero Gold to remove ribosomal RNAs and linear RNAs. Then these libraries were sequenced using the Illumina sequencing platform (HiSeq2500, Oebiotech Co. Ltd., Shanghai, China) and 150/125bp paired-end reads were generated.

Hisat2 and BWA software were used to align the clean reads of each sample with reference genome of the experimental specie. CIRI software was used to scan for PCC signals (paired chiastic clipping signals), and the prediction of circRNA sequences was performed based on junction reads and GT-AG cleavage signals. Differentially expressed transcripts were determined by Fold changes >2.0 or <0.5 and *p*-values < 0.05 between the two groups.

### Bioinformatics Analysis

Gene ontology (GO) enrichment analysis was applied to elucidate genetic regulatory network in terms of three aspects: biological processes, cellular components and molecular functions. The kyoto Encyclopedia of Genes and Genomes (KEGG) analysis was used to explore the biological pathways related to circRNA-targeting genes. To investigate the potential role of differentially expressed circRNAs, their host genes were input into the Database for Annotation, Visualization, and Integrated Discovery (DAVID6.8, http://david.abcc.ncifcrf.gov) for GO and KEGG analysis. Evaluation of the interactions between circRNAs and target miRNAs based on conserved seed-matching sequences was predicted by TargetScan algorithm (www.targetscan.org) and miRanda (www.microrna.org).

### Quantitative Real-Time Reverse Transcription Polymerase Chain reaction(qRT-PCR)

The extracted RNAs were reversely transcribed into cDNA using the PrimeScript RT Reagent Kit (Takara). Quantitative PCR (qPCR) was performed to verify differentially expressed circRNAs using SYBR Premix Ex Taq (TaKaRa) on an ABI7500 instrument. The PCR steps included initial denaturation at 95°C for 30 s, then 40 cycles at 95°C for 5 s, 60°C for 34 s. The amplified RNA products were stored at −20°C. Housekeeping gene GAPDH was detected as the internal control. Melt curves were conducted to check the product purity and the relative expression of target gene was determined by the 2^^(-△△Ct)^ method. The specific primers for the detected genes were listed in Supplementary Table S1.

### Enzyme-Linked Immunosorbent Assay (ELISA)

TNF-α, IL-1β, IL-10 and CCL22 levels in the supernatant of polarized macrophages were evaluated using ELISA Kits according to the manufacturer’s protocols. Cytokine concentrations (pg/ml) were determined by reading on a Bio-Rad (United States) Model 680 microplate reader at 415 nm. All of the antibodies’ kits were provided by R&D Systems, United States.

### Transfections

The siRNA transfection was performed according to the manufacturer’s instructions. Differentiated THP-1 macrophages were transfected with the synthesized small interfering RNA (siRNA) or Negative Control siRNA (Ruibo Guangzhou, China), using SuperFectin™ siRNA Transfection Reagent (Shanghai Pufei Biotech Co., Ltd., Shanghai, China), as recommended by the manufacturer. siRNA and transfection reagent were diluted, respectively with serum-free medium and then mixed. Final concentration of siRNA was 50 nmol/l per well. Non-transfected cells were analyzed simultaneously as a control. The sequence of circRNF19B siRNA was as follows: 5′-ACT​TGC​ATT​ACC​TCA​GTT​ATG-3′.

### Statistical Analysis

Numerical data were shown as the mean ± standard deviation (SD). GraphPad Prism 8 software was used to statistically analyze the differences of the data. Student’s t-test was applied for 2-group comparison and one-way ANOVA was applied for multiple-group comparisons. A value of *p* < 0.05 was considered to be statistically significant.

## Results

### Validation of THP-1 Derived Macrophages Polarization Phenotypes

**FIGURE 1:**
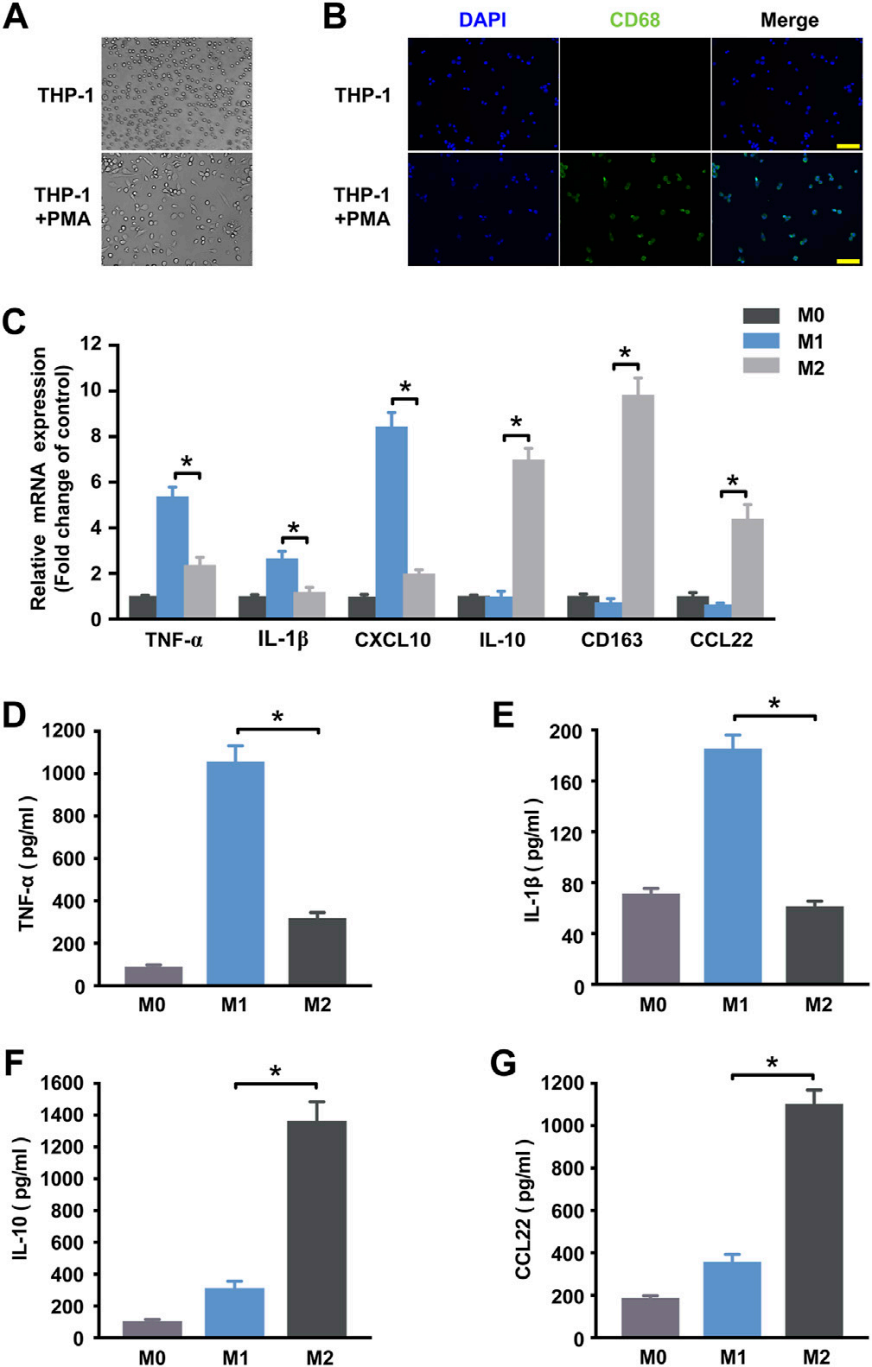
Determination of polarized M1 and M2 macrophages. **(A)** Induction of the macrophage-like phenotype in THP-1 cells. THP-1 cells were differentiated by PMA treatment for 48 h. Morphological changes were analyzed by comparing (up) undifferentiated cells versus (down) differentiated cells. Arrows indicate typical “spreading”. **(B)** Expression of the macrophage marker CD68 (green) in THP-1 cells at steady state (no treatment) and following 48 h of PMA stimulation. Nuclei were stained with DAPI (blue). Scale bar = 100 µm. **(C)** THP-1 macrophages treated with IFN-γ and LPS for M1 polarization or IL-4 for M2 polarization. The expression of cytokines of THP-1 macrophages was detected by qRT-PCR at 18 h post-treatment. M1 markers TNF-α **(D)** and IL-1β **(E)** and M2 markers IL-10 **(F)** and CCL22 **(G)** in the supernatant were detected by ELISA at 48 h post-treatment. Data are expressed as the means ± SD of at least three independent experiments (*n* = 4). *p* < 0.05, significant difference between M1 and M2 group.

Human THP-1 monocytic cell line, an immortal proliferating cell line, has been generally used to mimic the function and regulation of human primary macrophages. After exposure to phorbol 12-myristate 13-acetate (PMA), THP-1 cells can be differentiated into CD68^+^ macrophage phenotype (Figures 1A,B) (Qin 2012). In order to investigate alterations in the circRNAs expression profile during macrophage polarization, THP-1 macrophages were further differentiated into M1 macrophages by treating with IFN-γ and LPS and were differentiated into M2 polarization by treating with IL-4. qRT-PCR and ELISA analysis were performed to identify phenotypic and functional markers of M1 and M2 macrophages. Figure 1C showed that M1 macrophages expressed higher levels of TNF-α, IL-1β and CXCL10 than M2 macrophages, and consistently TNF-α and IL-1β were up-regulated in the supernatant of M1 macrophages (Figures 1D,E). While IL-10, CD163, and CCL22 mRNA expression levels in M2 macrophages (Figure 1C) and IL-10 and CCL22 protein levels (Figures 1F,G) of the supernatant were increased when compared to M1 macrophages. These results confirm that the incubation conditions used in THP-1-derived macrophages led to a well-characterized macrophage polarization.

### Overview of circRNA Expression Profiles

**FIGURE 2:**
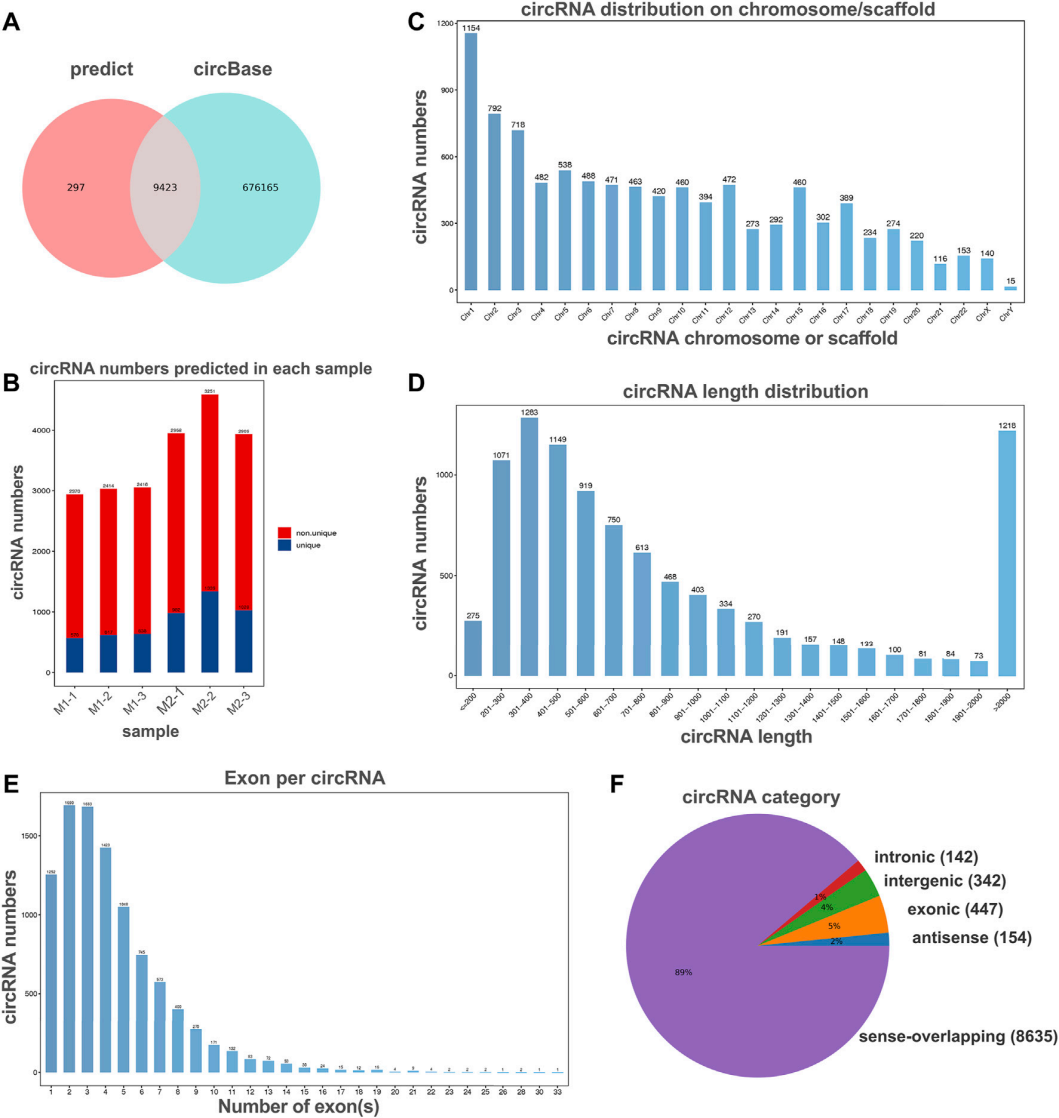
Overview of circRNA profiles of polaroized macrophages. **(A)** Venn analysis for comparison of predicted circRNAs with the data published in circBase. **(B)** The number of predicted circRNAs in M1 macrophages (M1-1, M1-2, M1-3) and M2 macrophages (M2-1, M2-2, M2-3). **(C)** Chromosome/scaffold distribution of predicted circRNAs. **(D)** Length distribution of predicted circRNAs. **(E)** The exon numbers distribution of predicted circRNAs. **(F)** Category of predicted circRNAs based on genomic origin.

To investigate the potential regulation functions of circRNAs in macrophage polarization, we explore the circRNA expression profiles in polarized THP-1 macrophages through next-generation RNA sequencing analysis. In total, 9,720 circular RNAs were detected in M1 and M2 macrophage group. By comparing with the data published in circBase, 9,423 of these circRNAs have been reported in circBase and the other 297 predicted circRNAs were novel (Figures 2A,B). These identified circRNAs originated from all the genome and the most circRNAs were transcribed from chromosomes 1, 2, and 3 (Figure 2C). The length of the circRNA candidates was maximum to 97,051 nucleotides (nt) and shortest to 89 nt, and the majority of them (8,572/9,720, 87.47%) were less than 2000 nt (Figure 2D). The number of exons per circRNA was less than seven for most circRNAs (8,415/9,720, 86.56%) (Figure 2E). According to the location of circRNAs in the genome relative to protein-coding genes, circRNAs can be divided into five categories, including 154 antisense, 447 exonic, 342 intergenic, 142 intronic, as well as 8,635 sense-overlapping circRNAs (Figure 2F). Obviously, sense-overlapping circRNAs were the most abundant, which suggests the importance of this kind of circRNA.

### Identification of Differentially Expressed circRNAs in Polarized Macrophages

**FIGURE 3:**
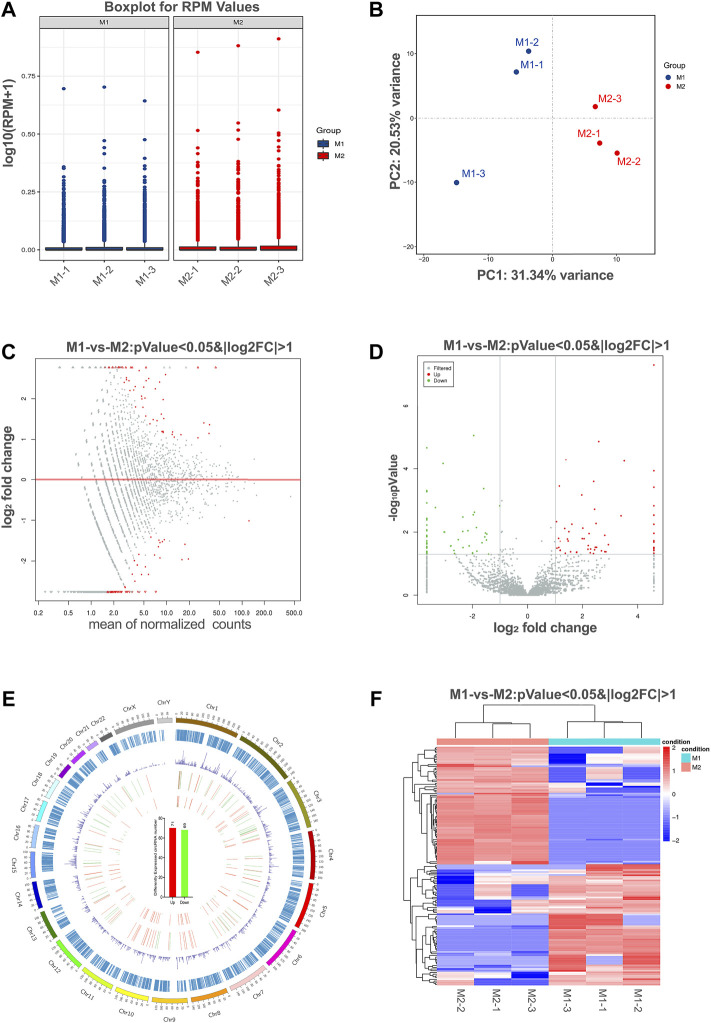
Differential expression of circRNAs in polarized macrophages. **(A)** Box plots of reads per million (RPM) values of circRNAs for the six samples after normalization. **(B)** Scatter plots of all samples for principal component analysis on the first principal component (PC1) and the second principal component (PC2). **(C)** MA scatter plots of sequencing data assessing overall distribution of the two sets of data. Labeled red portions indicate differentially expressed circRNAs (*p* < 0.05 and |log2FC|>1). **(D)** Volcano plot showing number and distribution of circRNAs in the same plane. **(E)** Chromosome distribution of up- and down-regulated circRNAs. **(F)** Heat map showing differentially expressed circRNAs from M1 macrophages compared with M2 macrophages. Each row represents one circRNA, and each column represents each sample. Red indicates up-regulation; blue indicates down-regulation.

The box plots showed the distribution of circRNA intensities after normalization, which suggested that no abnormal expression was observed in the six compared samples (Figure 3A). Principle component analysis showed samples in the same group distributed together (Figure 3B).

To identify the most significant candidates, raw data were normalized and converted into log2 values. A scatter plot was created to assess overall distribution of the two sets of data in a two-dimensional coordinate system (Figure 3C). Significant differentially expressed circRNAs were screened with absolute value of log2FC > 1 and *p* value <0.05. The number and distribution of circRNAs were shown in the volcano plot (Figure 3D). Furthermore, the differentially expressed circRNAs were clearly self-segregated into two groups through the hierarchical clustering analysis (Figure 3E). A total of 140 circRNAs were differentially expressed, including 71 circRNAs were up-regulated and 69 circRNAs were down-regulated in the M1 group when compared with M2 group (Figure 3F).

### Validation of Differentially Expressed circRNAs

**FIGURE 4:**
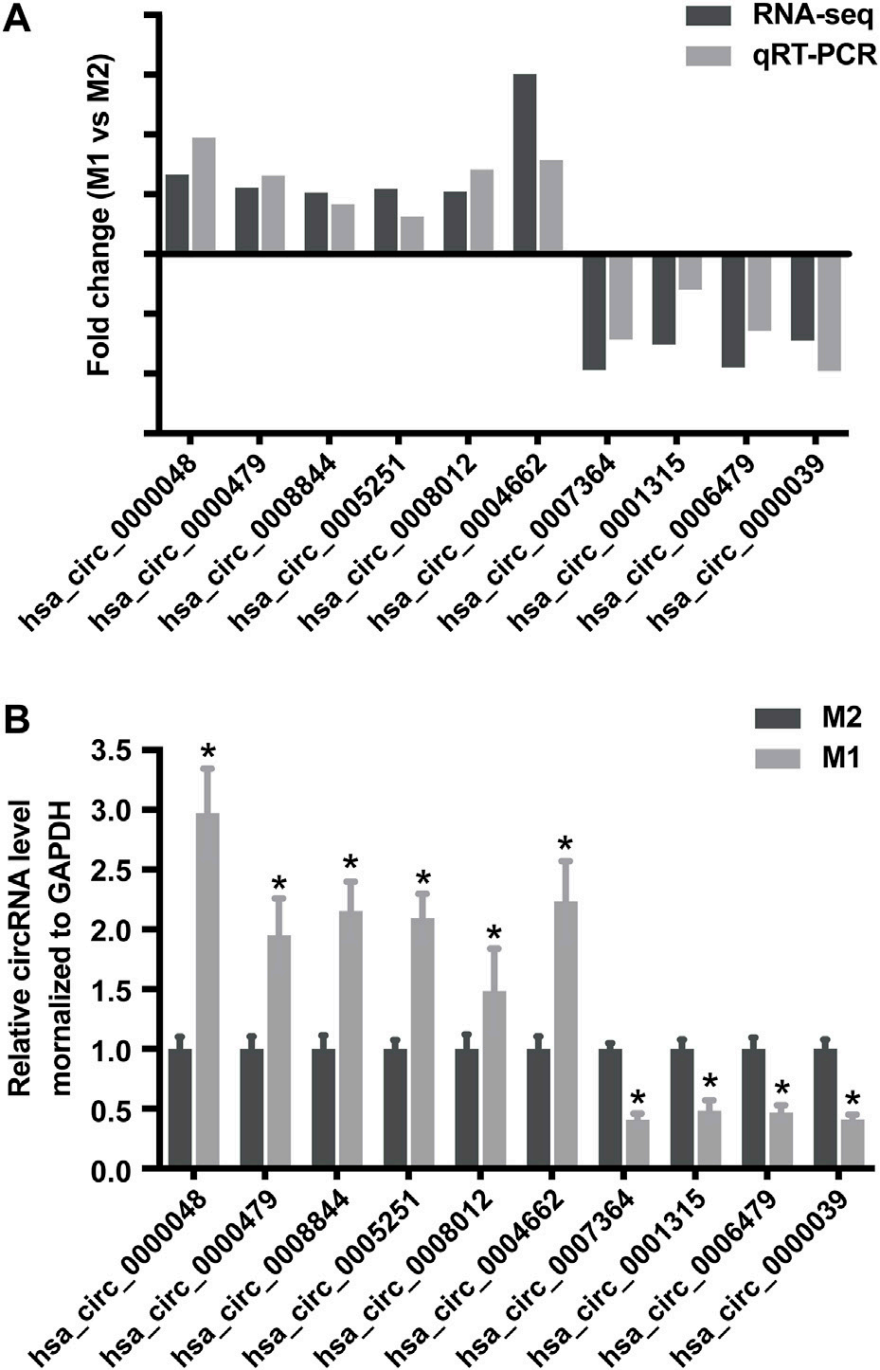
Validation of RNA sequencing data using qRT-PCR. **(A)** Compared with the expression of 6 up-regulated circRNAs and 4 down-regulated circRNAs using sequencing and qRT-PCR analysis between M1 and M2 macrophages. **(B)** Validation the expression of 6 up-regulated circRNAs and 4 down-regulated circRNAs in M1 macrophages compared with M2 macrophages. Data are expressed as the means ± SD of at least three independent experiments. *p* < 0.05, significant difference between M1 and M2 groups.

To validate the accuracy and reliability of sequencing data, 10 dysregulated circRNAs, including 6 up-regulated circRNAs (hsa_circ_0000048, hsa_circ_0000479, hsa_circ_0008844, hsa_circ_0005251, hsa_circ_0008012 and hsa_circ_0004662) and 4 down-regulated circRNAs (hsa_circ_0007364, hsa_circ_0001315, hsa_circ_0006479 and hsa_circ_0000039) were randomly selected for detection by qRT-PCR (Figures 4A,B; Table 1), which were roughly consistent with RNA sequencing results.

**TABLE 1:** Biological information regarding the 6 up- and 4 down-circRNAs in M1 macrophages.

CircRNA ID	FC (abs)	*p*-value	CircRNA type	Chromosome	Best_transcript	GeneSymbol
Up-regulation						
hsa_circ_0000048	2.6477035	5.15E-05	sense-overlapping	Chr1	XM_006710356.2	RNF19B
hsa_circ_0000479	2.2148449	0.0003506	sense-overlapping	Chr13	XM_011535312.2	EPSTI1
hsa_circ_0008844	2.0557745	0.0047254	sense-overlapping	Chr1	NM_001136493.2	MFSD2A
hsa_circ_0005251	2.1671681	0.0157849	sense-overlapping	Chr7	XM_011515590.2	FAM126A
hsa_circ_0008012	2.0793584	0.0319201	sense-overlapping	Chr4	XM_024454069.1	NFKB1
hsa_circ_0004662	6.0208777	1.35E-05	sense-overlapping	Chr6	NM_000636.3	SOD2
Down-regulation						
hsa_circ_0007364	3.8656042	8.69E-06	sense-overlapping	Chr1	NM_001195100.1	PTP4A2
hsa_circ_0001315	3.0166827	0.0004237	sense-overlapping	Chr3	NM_001112736.1	FAM208A
hsa_circ_0006479	3.7940778	0.0023380	ex1onic	Chr17	NM_001288733.1	TEX2
hsa_circ_0000039	2.8795207	0.0106659	exonic	Chr1	NM_001173128.1	YTHDF2

### Prediction of Biological Functions of Differentially Expressed circRNAs in M1 Macrophages

**FIGURE 5:**
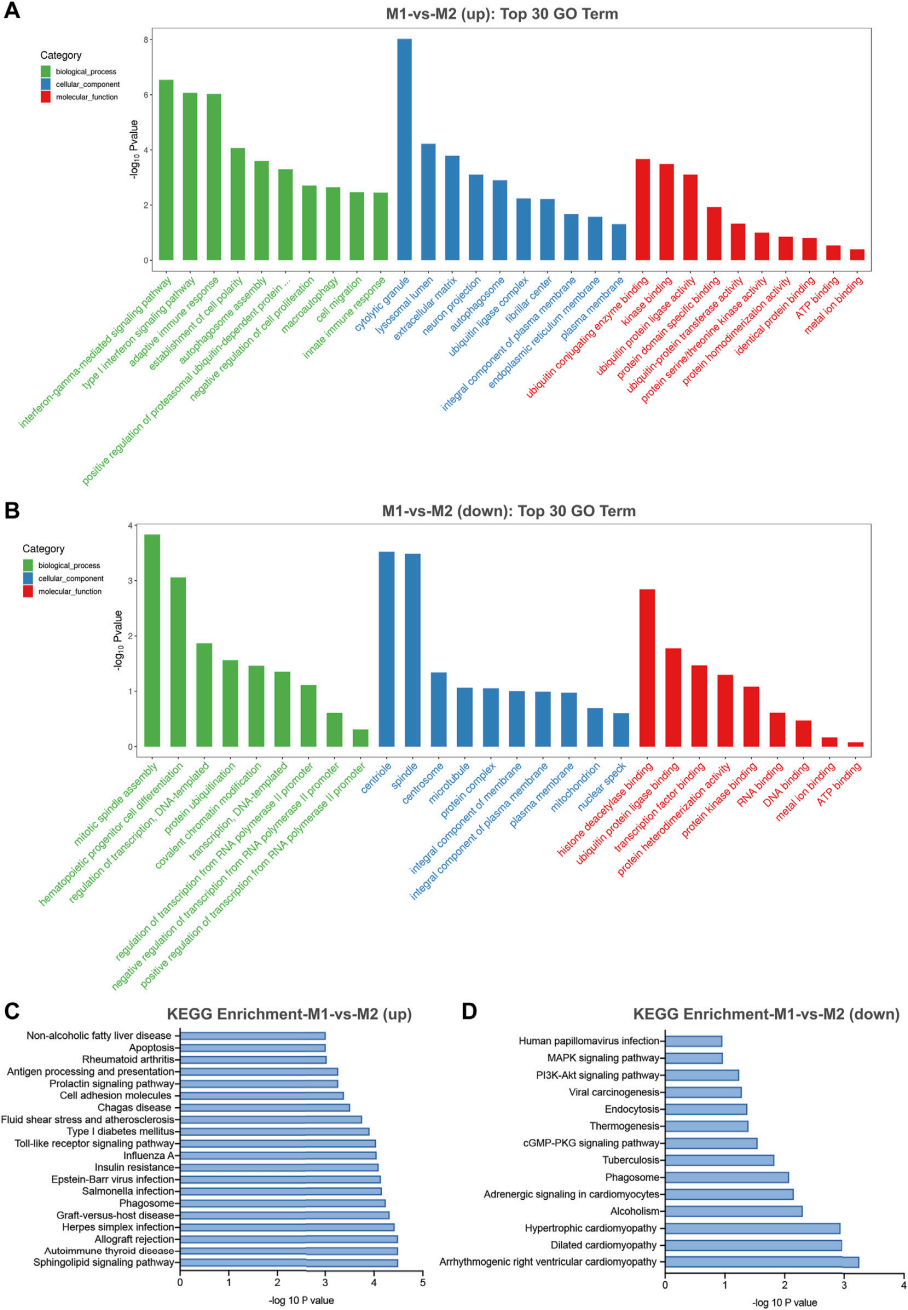
GO and KEGG pathway analysis for differentially expressed circRNAs in M1 macrophages. **(A)** The top 30 GO terms in biological process (BP), cellular component (CC) and molecular function (MF) categories for up-regulated circRNAs. **(B)** The top 30 GO terms in BP, CC and MF categories for down-regulated circRNAs. **(C)** The top 20 enriched significant enriched pathways for up-regulated circRNAs. **(D)** The top 14 enriched KEGG pathways for down-regulated circRNAs.

In general, circRNAs are generated from the exons or introns of their host gene. Evidence has shown that several circRNAs may serve to regulate the expression of their host genes (Barrett et al., 2015). Gene ontology (GO) and kyoto encyclopedia of genes and genomes (KEGG) pathway analysis of their host genes may contribute to predict the functions of these differentially expressed circRNAs. The most enriched GO terms in biologic process of up-regulated and down-regulated circRNAs were interferon-gamma-mediated signaling pathway (GO: 0060333) and mitotic spindle assembly (GO: 0090307) (Figures 5A,B). The most enriched GO term in cellular component of up-regulated and down-regulated circRNAs were cytolytic granule (GO: 0044194) and centriole (GO: 0005814) (Figures 5A,B). The most significant enriched GO term in molecular function of up-regulated and down-regulated circRNAs were ubiquitin conjugating enzyme binding (GO: 0031624) and histone deacetylase binding (GO: 0042826) (Figures 5A,B). Besides, KEGG pathway analysis showed the top pathways potentially involved in up- and down-regulated circRNA-mediated regulatory network, respectively. Among them, sphingolipid signaling pathway was the most significant associated pathway (Figures 5C,D).

### Annotation for circRNA-miRNA Interaction

**FIGURE 6:**
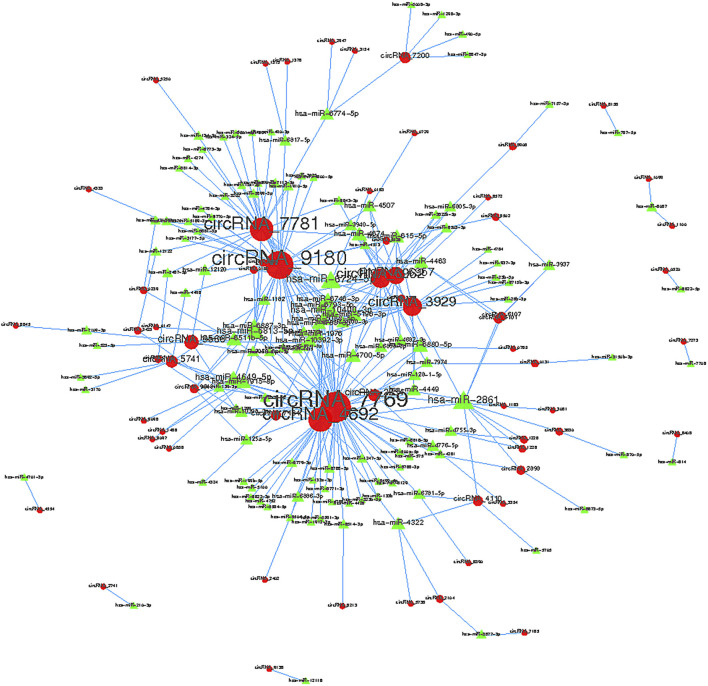
Top 300 predicted circular RNA-miRNA relationships. In the network, red nodes represent circRNAs, green inverted triangles represent the predicted miRNAs. and the edges represent sequence matching.

Collectively evidence has showed that circRNAs could function as miRNA sponges to compete for miRNA-binding sites, thus regulating target gene expression (Xu et al., 2015; Ebbesen et al., 2016; Salzman 2016). Cytoscape was used to screen out the top 300 circRNA-miRNA relationships according to the number of possible miRNA-binding sites, structure and energy score combined with *p*-value. The circRNA-miRNA network was consisted of 63 circRNAs and 133 miRNAs (Figure 6). As was shown in Figure 6, some circRNAs, such as hsa_circ_0005890 and hsa_circ_0115759, have many different predicted miRNAs binding sites and the other circRNAs, such as hsa_circ_0000099 and hsa_circ_0000133, have few predicted miRNA binding sites. Furthermore, the validated 6 up-regulated and 4 down-regulated circRNAs were selected, and the circRNA/miRNA interaction was predicted using the TargetScan and miRanda databases. The results pointed out that all 10 circRNAs could potentially bind to at least five miRNA response elements (Table 2). The above analysis suggests that the circRNAs could serve as miRNA sponges regulating gene expression in macrophage polarization.

**TABLE 2:** Annotation for differentially expressed circRNA/miRNA interaction.

CircRNA ID	GeneSymbol	MRE1	MRE2	MRE3	MRE4	MRE5
hsa_circ_0000048	RNF19B	miR-217	miR-6274-5p	miR-4691-3p	miR-6807-3p	miR-1207-3p
hsa_circ_0000479	EPSTI1	miR-942-5p	miR-5189-5p	miR-6833-3p	miR-7110-3p	miR-6809-3p
hsa_circ_0008844	MFSD2A	miR-100-3p	miR-1915-3p	miR-6764-5p	miR-6808-5p	miR-4673
hsa_circ_0005251	FAM126A	miR-30b-5p	miR-146a-3p	miR-3192-5p	miR-6509-3p	miR-301b-5p
hsa_circ_0008012	NFKB1	miR-3142	miR-1273g-5p	miR-196a-5p	miR-196b-5p	miR-5581-5p
hsa_circ_0004662	SOD2	miR-532-3p	miR-4520-2-3p	miR-6735-3p	miR-1199-5p	miR-135b-5p
hsa_circ_0007364	PTP4A2	miR-3609	miR-3614-5p	miR-6813-5p	miR-183-5p	miR-33a-3p
hsa_circ_0001315	FAM208A	miR-3686	miR-627-3p	miR-660-5p	miR-1288-3p	miR-181a-5p
hsa_circ_0006479	TEX2	miR-5193	miR-581	miR-1197	miR-149-3p	miR-877-3p
hsa_circ_0000039	YTHDF2	miR-324-3p	miR-29b-1-5p	miR-544b	miR-218-5p	miR-6515-5p

### The Involvement of circRNA-RNF19B in Regulating Macrophage Polarization

**FIGURE 7:**
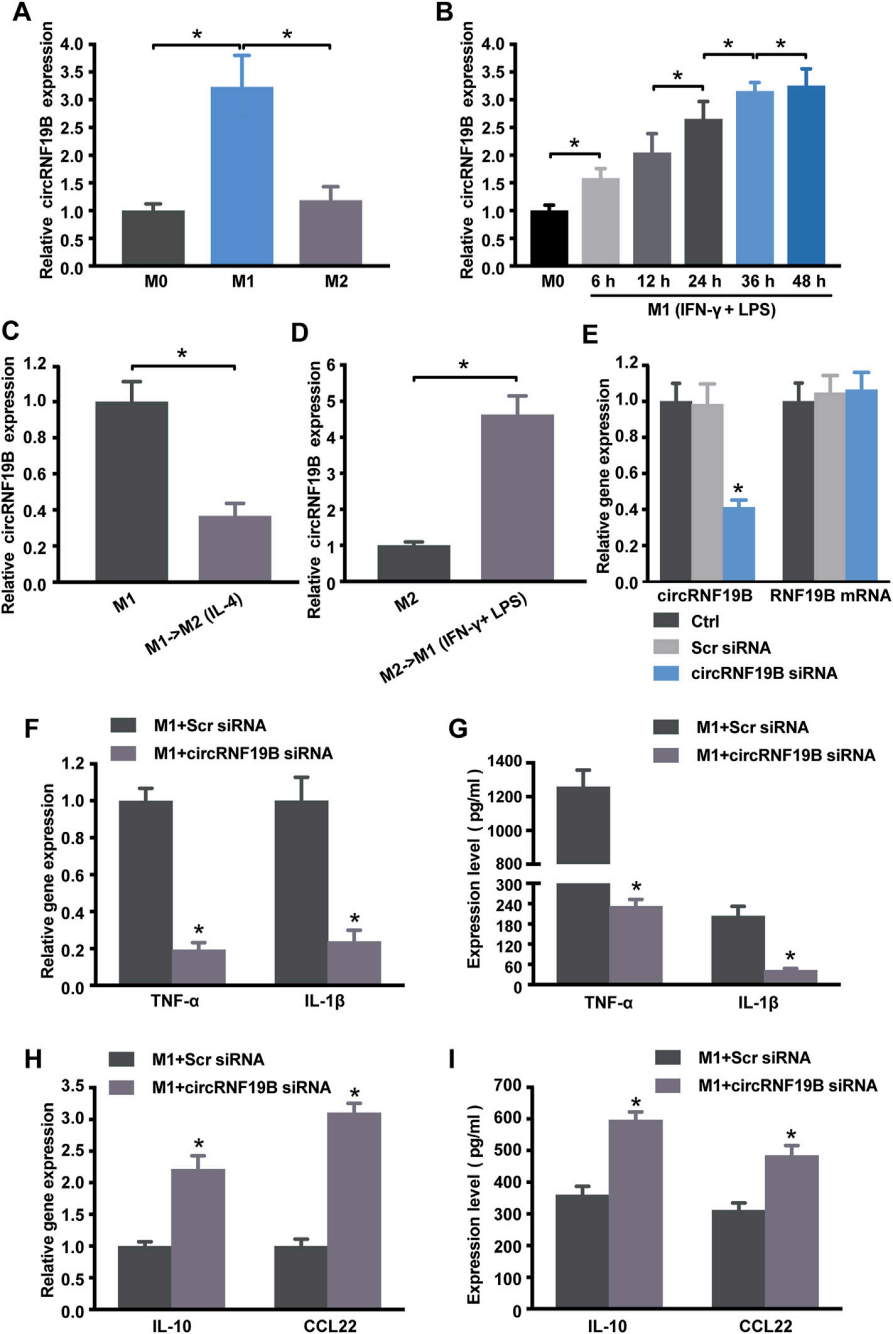
Regulation of circRNA-RNF19B in macrophage polarization. **(A)** THP-1 macrophages were treated with IFN-γ and LPS for M1 polarization or IL-4 for M2 polarization for 18 h or left untreated (M0). The expression of circRNF19B in each group was detected by qRT-PCR (*n* = 4). **(B)** THP-1 macrophages (M0) were treated with IFN-γ and LPS for M1 polarization for the indicated time points. qRT-PCRs were conducted to detect circRNF19B expression (*n* = 5). **(C)** qRT-PCRs were conducted to detect circRNF19B expression levels in macrophages following M1-to-M2 re-polarization by IL-4 for 18 h (*n* = 4). **(D)** qRT-PCRs were conducted to detect circRNF19B expression levels in macrophages following M2-to-M1 re-polarization by IFN-γ plus LPS treatment for 18 h (*n* = 4). **(E)** THP-1 macrophages were transfected with scrambled (Scr) siRNA, siRNA targeting the sequence of circRNF19B, or left untreated (Ctrl) for 36 h qRT-PCRs were conducted to detect circRNF19B and RNF19B mRNA expression (*n* = 4, *p* < 0.05 versus Scr siRNA). THP-1 macrophages were transfected with circRNF19B siRNA or Scr siRNA and then stimulated with LPS and IFN-γ for M1 polarization. M1 markers TNF-α and IL-1β **(F)** and M2 markers IL-10 and CCL22 **(H)** in each group were detected by qRT-PCR at 18 h post-treatment. M1 markers TNF-α and IL-1β **(G)** and M2 markers IL-10 and CCL22 **(I)** in the supernatant of each group were detected by ELISA at 48 h post-treatment (*n* = 5, *p* < 0.05 versus the corresponding Scr siRNA). Data are expressed as the means ± SD of at least three independent experiments. *p* < 0.05, significant difference between the two groups compared.

As shown in Figure 4, the sequencing data and validated qRT-PCR results both showed that hsa_circ_0000048 was remarkably up-regulated in M1 macrophages compared to M2. Hsa_circ_0000048 is derived from the RNF19B gene exon 2-3, with a spliced mature sequence length of 351 bp. Furthermore, RNF19B was reported to be involved in macrophage inflammatory cytokine production (Lawrence, et al., 2015; Lawrence, et al., 2020). Hence, to further explore the functional roles of circRNA in macrophage polarization, we selected circRNF19B as a candidate circRNA for further investigation. The results showed that circRNF19B expression was significantly up-regulated in M1 macrophages compared with THP-1 macrophages (M0) and M2 macrophages (Figure 7A). What’s more, the expression of circRNF19B in macrophages increased gradually with the prolongation of IFN-γ and LPS stimulation (Figure 7B), while the expression of circRNF19B in IL-4 stimulated macrophages didn’t significantly change (Supplementary Figure S1A).

In order to investigate the expression changes of circRNF19B during macrophage polarization transition process, we used IL-4 to induce M1 macrophage repolarization to M2 (Supplementary Figure S1B), and IFN-γ and LPS induced M2 macrophage repolarization to M1 phenotype (Supplementary Figure S1C). The expression of circRNF19B was decreased following M1-to-M2 phenotype transition (Figure 7C). Conversely, the expression of circRNF19B was increased following M2-to-M1 phenotype transition (Figure 7D). Thereafter, circRNF19B siRNA and a control oligonucleotide (Scr siRNA) were transfected into THP-1 macrophages, which were following induced to polarize to M1 phenotype. The results showed that circRNF19B expression was considerably down-regulated but didn’t affect the expression of its host gene (Figure 7E). Knockdown of circRNF19B successfully re-polarized M1 macrophage to M2 phenotype as shown by decreased NF-α and IL-1β (Figures 7F,G), and increased IL-10 and CCL-22 (Figures 7H,I). These findings suggest that circRNF19B is involved in regulating macrophage polarization.

## Discussion

Macrophages are heterogeneous which can be roughly induced to two diverse phenotypes respond to different external stimuli. Functional skewing of macrophages occurs not only under physical conditions such as ontogenesis and pregnancy but also in pathological situation such as inflammation, metabolic diseases and immunity (Shapouri-Moghaddam et al., 2018). Progress has been made in exploring the molecular networks and mechanisms underlying macrophage polarization, which include members of NF-κB, STAT, sphingolipid signaling pathway and KLF families (Sica and Mantovani 2012; Locati et al., 2020). However, the corresponding stimulating factors and precise regulation mechanism have not been clearly stated. In this study, IFN-γ/LPS stimulated THP-1 macrophages to induce M1-type polarization, and IL-4 stimulated macrophages to induce M2-type polarization. These data are in accordance with previous studies (Genin et al., 2015; Maess et al., 2015; Bras et al., 2017). Based on the successful construction of the polarization model, next-generation RNA sequencing analysis was used for demonstration of the circRNAs expression profiling in distinct polarizing macrophages. 140 circRNAs were found to be abnormally expressed in M1 macrophages as compared with M2. The expression of hsa_circ_0000048 (circRNF19B) in M1 phenotype was greatly higher than that in M2. It is further confirmed that circRNF19B was involved in promoting macrophages polarization to M1 phenotype, suggesting a promising potential target for treating macrophage polarization-related diseases.

CircRNAs are a class of particular non-coding RNA molecules without any free terminal and are usually derived from the exonic or intronic sequences. They can regulate gene expression through a variety of molecular mechanisms including interacting with RNA-binding proteins, impacting splicing, acting as regulators of parental gene expression or microRNA sponges (Lasda and Parker 2014; Huang et al., 2017). Therefore, it is possible to predict the function of some circRNAs by means of their parental gene function. GO enrichment analysis was conducted to explore the function of host genes of differently expressed circRNAs in macrophage polarization. Among the GO terms, interferon-gamma-mediated signaling pathway is one of the basic biologic behaviors underlying the polarization of macrophages (Mead et al., 2003; Liu and Wang 2021). The most significant enriched GO term in molecular function of up-regulated and down-regulated circRNAs were ubiquitin conjugating enzyme binding and histone deacetylase binding. Arora and colleagues demonstrate that ABCF1 is an E2 ubiquitin-conjugating enzyme that regulates macrophage polarization to dampen lethal septic shock (Arora et al., 2019). SIRT1, a member belonging to the class III deacetylase family, and histone deacetylase 9 (HDAC9) play important roles in balancing M1/M2 macrophages (Zhang et al., 2018) and vascular inflammation (Arora et al., 2019).

Pathway analysis was focused on sphingolipid signaling pathway which may potentially take part in circRNA-mediated regulatory network. Interestingly, two lysosphingolipids, sphingosylphosphorylcholine (SPC) and lysosulfatide (LSF), have an inverse effect on macrophagic differentiation of monocytic cell lines, U937 and THP-1 (Yamamoto et al., 2011). Sphingosine-1-phosphate (S1P), another bioactive lysosphingolipid, is generated by phosphorylation of sphingosine in response to an enzyme family consisting of sphingosine kinase (SPHK)1 and 2 (Weigert et al., 2009). It has been reported that the role of S1P on macrophage phenotype can decisively influence the emergence of pathological states, most notably inflammatory diseases (Weigert et al., 2009; Ji et al., 2020). Extracellular S1P provokes an anti-inflammatory macrophage phenotype by inhibiting the production of pro-inflammatory cytokines and activation of NF-kB, while enhancing the expression of anti-inflammatory and pro-angiogenic mediators. In the rat model of acute necrotizing pancreatitis (Liu et al., 2008), S1P alleviates acute pulmonary inflammation and injury through inhibiting NF-κB activity in alveolar macrophages associated with decreased production of IL-1β, IL-6 and TNF-α. Furthermore, recent findings suggest that S1P may selectively attenuate Toll-like receptor 2 signaling via negative cross-talk between S1P1/2 and toll-like receptor 2 signaling pathways in murine macrophages, thus preventing the activation of inflammatory macrophage (Dueñas et al., 2008). Based on the above evidence, we speculate that circRNA-mediated regulatory networks are involved in the polarization process of macrophages.

Accumulated evidence show that circRNAs carry binding sites for miRNAs of interest and inhibit their function (Hansen et al., 2013; Panda 2018). miRNAs control several aspects of gene regulation through direct interaction with their target mRNA (Ali Syeda et al., 2020). As a result, circRNAs could derepress the canonic targets of corresponding miRNAs. CDR1 is shown to harbor more than 70 sites for miR-7 binding (Memczak et al., 2013). Hsa_circ_0076690 is identified to act as a sponge of miR-152 and regulate osteogenic differentiation of hBMSCs (Han et al., 2020). Our bioinformatics analysis showed the circRNA/miRNA interaction for the differently expressed circRNAs in M1 macrophages. Among them, 10 circRNAs contained at least five miRNA binding sites. What’s more, data are widely available about the role of miRNAs in macrophage polarization (Wu et al., 2016; Self-Fordham et al., 2017). Thus, it is not surprising that circRNA-miRNA-mRNA network is potentially involved in macrophage polarization-related processes. Naturally, the regulatory mechanisms under that network and the function of their interaction will be the focus of our future research.

Increasing studies have illustrated that ncRNAs take part in modulating macrophage polarization. Most studies have focused on the role of miRNAs and lncRNAs in shaping macrophage polarization and plasticity (Huang et al., 2016; Tran et al., 2016; Li et al., 2018; Zhou et al., 2018). Reportedly, miR-511-3p could control macrophage polarization and shape the immune response by inhibiting its direct targets (e.g., PTGDS, ROCK2, and LTBP1) or regulating the expression of indirect targets (e.g., TLR4 and C/EBPα) (Zhou et al., 2018) and miR-223 has been shown to promote plasticity from the M1 to M2 phenotype and reduce the production of pro-inflammatory cytokines (Tran et al., 2016). Recently, lncRNA-CMPK2 and THRIL are found to involve in macrophages response to inflammatory conditions. LncRNA-TCONS_00019715 is identified to promote THP-1 macrophages polarization to M1 phenotype (Huang et al., 2016). Furthermore, evidence shows that lncRNA TUC339 expression is positively correlated with M2 macrophage polarization (Li et al., 2018). Here, we found that circRNF19B was strongly induced under M1 polarizing condition. To detect whether circRNF19B contribute to regulating macrophage polarization, we attempted to convert one phenotype into the other by culturing M2 macrophages with IFN-γ/LPS or M1 macrophages with IL-4. The results demonstrated that M2-to-M1 conversion led to increased circRNF19B expression, while M1-to-M2 resulted in decreased circRNF19B expression. It was intriguing that knockdown of circRNF19B diminished the polarization to M1 accompanied by the elevated expression of M2 markers. These findings suggested that circRNF19B might be an important factor involved in modulating macrophage plasticity and promoting macrophage polarization to the M1 phenotype.

In summary, the present study has investigated the global expression patterns of circRNAs in different macrophage polarization and provides information beneficial to a better understanding of macrophage functional activity from the perspective of circRNAs. Moreover, we found that circRNF19B could facilitate macrophages converting to the M1 phenotype. In the future, further studies are warranted to investigate the correlation between the circRNAs identified in sequencing analysis and macrophage differentiation and function. Moreover, further studies are also required to elucidate the mechanism underlying circRNA-mediated macrophage polarization.

## Data Availability

The datasets presented in this study can be found in online repositories. The names of the repository/repositories and accession number(s) can be found below: https://www.ncbi.nlm.nih.gov/bioproject/, PRJNA781473.
