# Supplementary material for: Comparative Analysis of Differentially Expressed Circular RNAs in Polarized Macrophages
Source: Front Genet. 2022 Mar 16;13:823517. doi: 10.3389/fgene.2022.823517 (PMC8967150; doi:10.3389/fgene.2022.823517)
Supplement: Supplementary file 1 [file DataSheet1.docx]

**Supplementary** **Figure 1. Regulation of circRNA-RNF19B in macrophage polarization.**

**
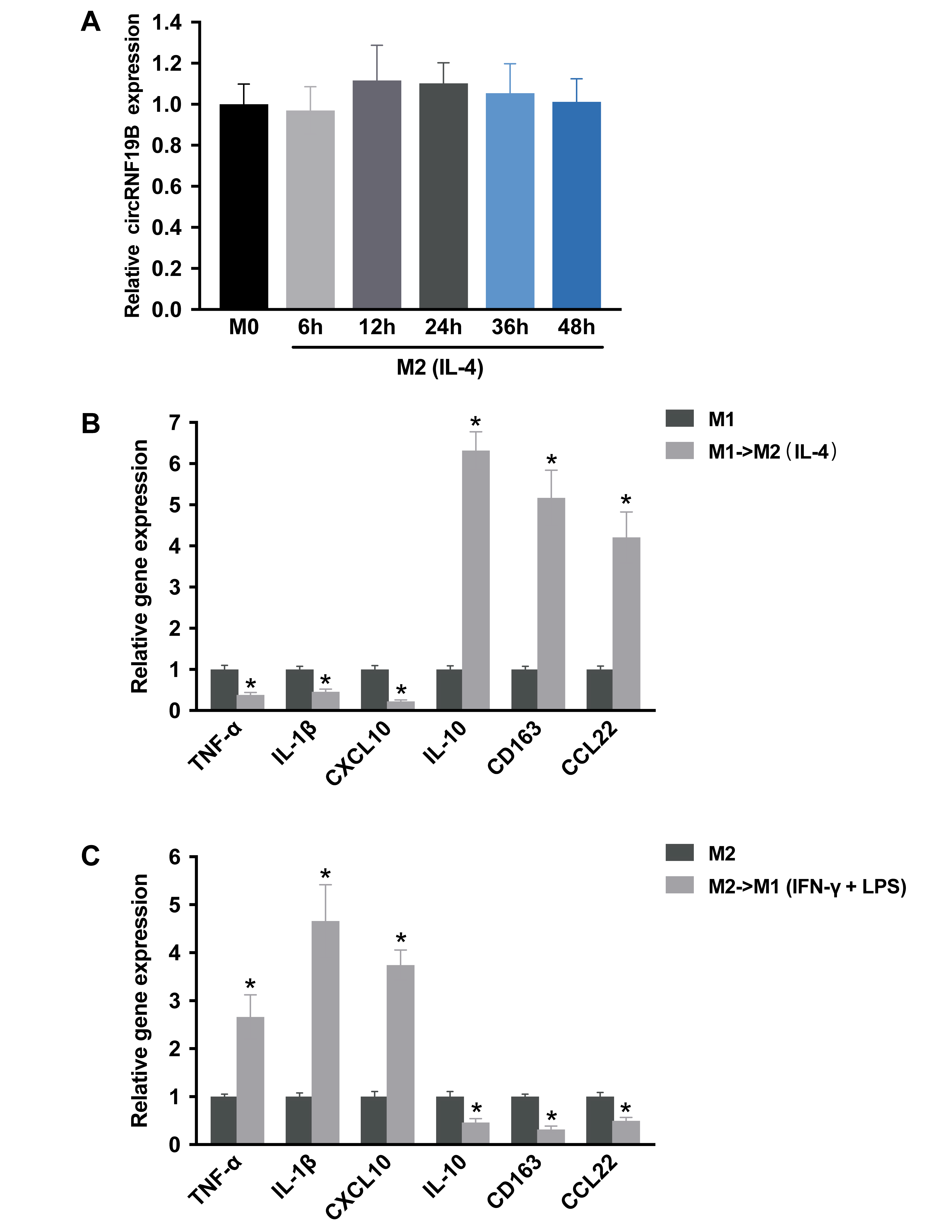
**

(A) THP-1 macrophages (M0) treated with IL-4 for M2 polarization for the indicated time points. qRT-PCRs were conducted to detect circRNF19B expression (n=5). (B) qRT-PCRs were conducted to detect cytokine expression levels in macrophages following M1-to-M2 re-polarization by IL-4 for 18h (n=4). (C) qRT-PCRs were conducted to detect cytokine expression levels in macrophages following M2-to-M1 re-polarization by IFN-γ plus LPS for 18h (n=4). *p* < 0.05, significant difference between M1 and M2 group.

**Supplementary Table 1. Sequences of qRT-PCR Primers.**

| Gene Symbol | Primer | (5’-3’) |
| --- | --- | --- |
| circRNF19B | Forward  Reverse | GATGAATGATGGAAGCTGTAATCAC  GGCAATAACAGCATAACTGAGGT |
| RNF19B | Forward  Reverse | AAGCCAAACCAAGCCACTATC  TCTCTGCCATCTGAGCATGAAC |
| TNF-α | Forward  Reverse | TATGGAGACAGATGTGGGGTG  CTTAGCCCTGAGGTGTCTGG |
| IL-1β | Forward  Reverse | TGCTCAAGTGTCTGAAGCAG  TGGTGGTCGGAGATTCGTAG |
| IL-10 | Forward  Reverse | GACTTTAAGGGTTACCTGGGTTG  TCACATGCGCCTTGATGTCTG |
| CXCL10 | Forward  Reverse | GAACCTCCAGTCTCAGCACC GAGAGGTACTCCTTGAATGCCA |
| CD163 | Forward  Reverse | CAGTGAGTTCAGCCTTTAAGATACC  GAATCCTAGGAGAAGAAGAACTCCA |
| CCL22 | Forward  Reverse | ATTACGTCCGTTACCGTCTG  TAGGCTCTTCATTGGCTCAG |
| GAPDH | Forward  Reverse | GAACGGGAAGCTCACTGG  GCCTGCTTCACCACCTTCT |
| hsa_circ_0000479 | Forward  Reverse | CATTTAGAGAGCATCAGCAATACAC  CTCTGTTCTGCTCCTTCCACTTC |
| hsa_circ_0008844 | Forward  Reverse | CGCCTTATGCCCTGAAAGAAC  AGGAAGAAACCCAGGGCACA |
| hsa_circ_0005251 | Forward  Reverse | GCCTTAACTTACATGCCCAGTG  CCATTCCTCCACAACCCCTTT |
| hsa_circ_0008012 | Forward  Reverse | GCCCATCCCATGGTGGACTA  GAAACATTTGTTCAGGCCTTCCC |
| hsa_circ_0004662 | Forward  Reverse | GCTTGCAAAAAGTAAACCACG  TTAGGGCTGAGGTTTGTCCAG |
| hsa_circ_0007364 | Forward  Reverse | GGAGTGACGACTTTGGTTCG  TGTCAGCGAAAATGCTGTGC |
| hsa_circ_0001315 | Forward  Reverse | TGCAGCCTTTATGAAGTTGTGG  ATTCTCGAGAGCCTGGAGTT |
| hsa_circ_0006479 | Forward  Reverse | ATCTGCGACACTGGAACACA  TGTCAGTGGTTTTCTCGGCA |
| hsa_circ_0000039 | Forward  Reverse | TATAGCCAGCTACAAGCACAC  AAGCCAATGGAGGGACTGTA |
